# Unloading in Refractory Cardiogenic Shock After Out-Of-Hospital Cardiac Arrest Due to Acute Myocardial Infarction—A Propensity Score-Matched Analysis

**DOI:** 10.3389/fcvm.2021.704312

**Published:** 2021-08-24

**Authors:** Jan-Thorben Sieweke, Muharrem Akin, Julian-Arman Beheshty, Ulrike Flierl, Johann Bauersachs, Andreas Schäfer

**Affiliations:** Department of Cardiology and Angiology, Cardiac Arrest Center and Advanced Heart Failure Unit, Hannover Medical School, Hanover, Germany

**Keywords:** cardiogenic shock, left ventricular unloading, myocardial infarction, out of hospital cardiac arrest, culprit lesion

## Abstract

**Aims:** Unclear neurological outcome often precludes severely compromised patients after out-of-hospital cardiac arrest (OHCA) from mechanical circulatory support (MCS), while it may be considered as rescue therapy for patients with refractory cardiogenic shock (rCS) in the absence of OHCA. This analysis sought to investigate the role of left ventricular (LV) unloading in patients with rCS related to acute myocardial infarction (AMI) after OHCA.

**Methods:** Of 273 consecutive patients receiving microaxial pumps in the Hannover Cardiac Unloading Registry between January 2013 and August 2018, 47 presented with AMI–rCS following successful resuscitation. Subsequently, the patients were compared by propensity score matching to patients with OHCA AMI–rCS without MCS. The patient data for OHCA without LV unloading was available from 280 patients of the Hannover Cooling Registry for the same time period. Furthermore, the patients with OHCA without rCS were compared to the patients with OHCA AMI–rCS and LV unloading.

**Results:** In total, 15 OHCA AMI–rCS patients without MCS were matched to patients with AMI–rCS and Impella. Patients without LV support had a higher proportion of a cardiac cause of death (*n* = 7 vs. *n* = 3; *p* = 0.024). LV unloading with Impella counteract rCS status and was associated with a preferable 30-day survival (66.7 vs. 20%, *p* = 0.01) and a favorable neurological outcome after 30 days (Cerebral Performance Category ≤2, 47 vs. 27%). Impella support is associated with a higher 30-day survival (odds ratio, 2.67; 95% confidence interval, 1.02–13.66).

**Conclusion:** In patients after OHCA with AMI–rCS, Impella support incorporated in a strict standardized treatment algorithm results in a preferable 30-day survival and counteracts severe rCS status.

## Introduction

Acute myocardial infarction (AMI) is a main contributor to out-of-hospital cardiac arrest (OHCA) (1). Despite improvements in diagnosis and treatment, the mortality rates remain high (2). Most patients suffer from post-cardiac arrest syndrome characterized by reduced systemic perfusion due to vasoplegia and adverse metabolism. Therefore, the early recovery of systemic perfusion to prevent end-organ dysfunction is relevant (3), and cardiac revascularization is recommended (1).

In patients with AMI complicated by cardiogenic shock (CS), percutaneous coronary intervention (PCI) of the culprit artery reduced mortality (4, 5). However, despite PCI, decreased cardiac output and metabolic deterioration contribute to end-organ failure, itself leading to a vicious cycle resulting in mortality (6).

Therefore, several percutaneous mechanical circulatory support (MCS) devices attracted attention to rescue patients in refractory cardiogenic shock (rCS) and are recommended by current guidelines (7, 8). In hemodynamically severely compromised patients, the Impella microaxial flow-pump, percutaneously inserted *via* a femoral approach, actively unloads the left ventricle independent of intrinsic left ventricular (LV) function, with the consequence of reduced wall tension and ventricular dimension. The Impella increases myocardial perfusion while maintaining cardiac output and improving end-organ perfusion (9, 10).

However, due to the lack of prospective randomized trials and conflicting results, the efficacy of active LV unloading in patients with OHCA complicated by AMI–rCS has not been determined yet.

We previously demonstrated that an early treatment algorithm (Hannover Cardiac Resuscitation Algorithm, HaCRA) with a multidisciplinary approach, including therapeutic hypothermia, coronary revasularization, and hemodynamic support, in rCS patients after OHCA is associated with lower mortality as described before (11).

Therefore, this analysis sought to investigate whether active LV unloading with Impella in patients after OHCA with AMI–rCS imbedded in a dedicated early in-hospital algorithm (HaCRA) is associated with a preferable outcome.

## Methods

### Study Design and Participants

The HAnnover Cardiac Unloading REgistry (HACURE) has a prospective and observational design. The HACURE includes all consecutive patients who received an Impella microaxial pump for LV unloading in our department. The HACORE includes all patients admitted after out-of-hospital cardiac arrest and receiving therapeutic hypothermia as part of a standard treatment at the cardiac arrest center at Hannover Medical School. All patients in both registries were treated according to HaCRA. The current analysis is in accordance with the Declaration of Helsinki and approved by the ethics committee at Hannover Medical School (#3566-2017).

**Figure 1:**
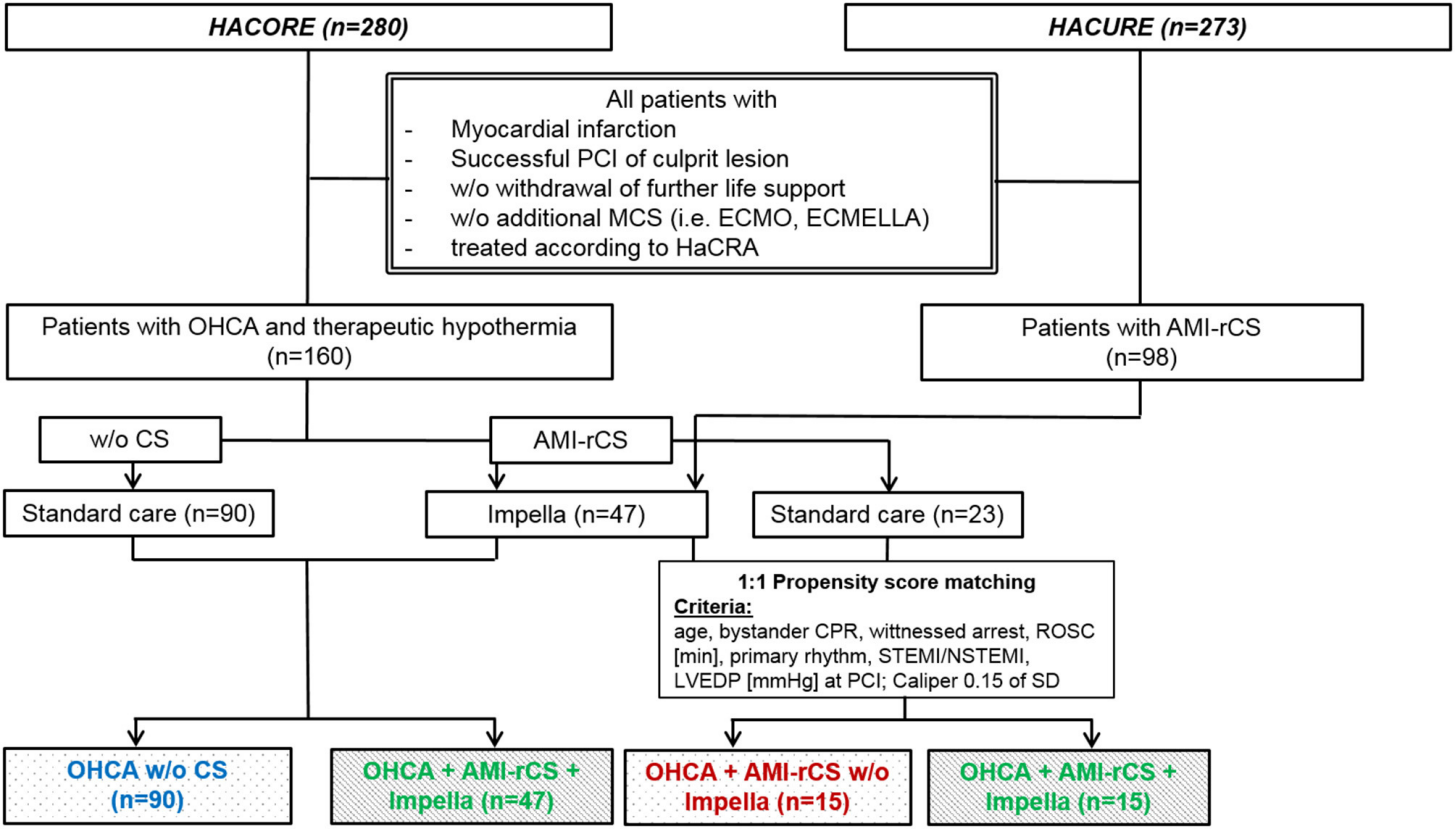
Study enrolment. AMI, acute myocardial infarction; CPR, cardio-pulmonary resuscitation; CS, cardiogenic shock; ECMELLA, circulatory support by a combination of Impella and ECMO; ECMO, extracorporeal membrane oxygenation; HACORE, Hannover Cooling Registry; HaCRA, Hannover Cardiac Resuscitation Algorithm; HACURE, Hannover Cardiac Unloading Registry; MCS, mechanical circulatory support; NSTEMI, non-ST elevation myocardial infarction; PCI, percutaneous intervention; OHCA, out-of-hospital cardiac arrest; rCS, refractory cardiogenic shock; ROSC, return of spontaneous circulation; SD, standard deviation; STEMI, ST elevation myocardial infarction; w/o, without.

We analyzed consecutive patients after OHCA with AMI (either ST segment elevation myocardial infarction or non-ST segment elevation myocardial infarction) and successful PCI of the culprit lesion, complicated AMI–rCS treated with MCS using Impella, and mandatory therapeutic hypothermia who were admitted to the Department of Cardiology at Hannover Medical School between January 2013 and August 2018. In the current analysis, the exclusion criteria were defined as follows: patients without myocardial infarction, mechanical cause of rCS, withdrawal of further life support, isolated right ventricular or biventricular failure at baseline, use of additional MCS (i.e., extracorporeal membrane oxygenation) or unidentifiable culprit lesion or unsuccessful PCI of the culprit lesion. Consecutive patients from the HAnnover COoling REgistry (HACORE, *n* = 280) and HACURE (*n* = 273) were defined as controls and allocated into groups as follows: (1) patients with AMI following OHCA in the absence of CS (*n* = 90) and (2) patients with AMI following OHCA and complicated by rCS without MCS (*n* = 23). Subsequently, to analyze the impact of circulatory support in patients with AMI–rCS after OHCA, a propensity score (PS) matching was considered (OHCA+AMI–rCS without Impella *vs*. OHCA + AMI–rCS + Impella). Furthermore, to verify the applicability of HaCRA to patients after extrahospital resuscitation with refractory cardiogenic shock and support with an Impella, we compared the patients after OHCA without CS and the patients with OHCA AMI–rCS and active LV support by Impella. To avoid unmeasured confounding, these cohorts were not considered for PS matching as described in Figure 1. The endpoints were defined as follows: The primary endpoint of this analysis was 30-day mortality in the PS-matched cohorts. The secondary endpoint was defined as 30-day mortality in the group of patients with AMI–rCS and Impella support and patients without CS. Furthermore, the endpoints for the safety outcome in all cohorts are as follows: peripheral ischemic complications forcing vascular surgery or intervention, mild/moderate/severe bleeding assessed by GUSTO, and neurological outcome after 30 days of admission as assessed by cerebral performance category (CPC). We defined a good neurological outcome as CPC ≤2, as previously described (12). The detailed study design is provided in the Supplementary Material.

### Patient Treatment and Definitions

The patients were treated according to current guidelines (8, 13, 14) and a standardized multidisciplinary local treatment algorithm, HaCRA, for CS and cardiac arrest as previously described (11). Details on patient treatment and clinical follow-up are provided in the Supplementary Material.

### Statistical Analysis

The data were analyzed using GraphPad Prism 7.04 (GraphPad Software, San Diego, CA, USA), R program 3.3.3, and SPSS 25 (IBM SPSS Statistics 25). The categorical parameters are presented as counts and percentages. The metric normally distributed variables are presented as mean values ± standard deviation and the non-normally as median and interquartile ranges. Normality and variance homogeneity were checked by Shapiro–Wilk and Kolmogorov–Smirnov tests, respectively. The statistical analysis for comparison between PS-matched groups of metric parameters was performed using unpaired *t*-tests as parametric tests and Mann–Whitney tests as non-parametric tests. Chi-square test was applied to compare nominally scaled parameters. In the PS-matched groups, there was no missing data for the documented parameters. The 30-day survival was calculated using Kaplan–Meier curves, and log-rank comparison was performed between the groups. Cox regression analysis was performed to calculate hazard ratios (HR) with 95% confidence intervals (CI). The reported *P*-values are two-sided, with *p* < 0.05 considered statistically significant.

### Propensity Score Matching

To minimize confounder bias and realize a balanced distribution of baseline characteristics to estimate the effects of MCS with Impella in patients after OHCA and AMI complicated by rCS, a PS matching was performed to patients in the control cohort as described above. The propensity scores were estimated using multivariable logistic regression modeling accounting for variables related to the outcome or which are clinically meaningful: age, bystander CPR, witnessed cardiac arrest, ROSC, primary rhythm, STEMI/NSTEMI (11), and LVEDP at the time of PCI. The cases and control groups were matched stepwise on the logit of the estimated propensity score (1:1 propensity score matching) using a nearest-neighbor model using calipers with the width equal to 0.15. A lower caliper width was used to maximize correct matching and to reduce bias.

The baseline balance of parameters used for the matching between patients after OHCA with AMI and successful PCI of the culprit lesion, complicated by rCS treated with MCS using Impella and comparators before and after PS matching, was compared *via* a standardized difference (15). A standardized difference ≤0.15 suggested an appropriate balance between the covariates (Supplementary Table 1). To validate the method and perform a sensitivity analysis of the propensity score matching, the primary outcome (30-day survival) was reanalyzed using the entire (unmatched) cohort (Supplementary Figure 1).

## Results

### Patient Characteristics

**Figure 2:**
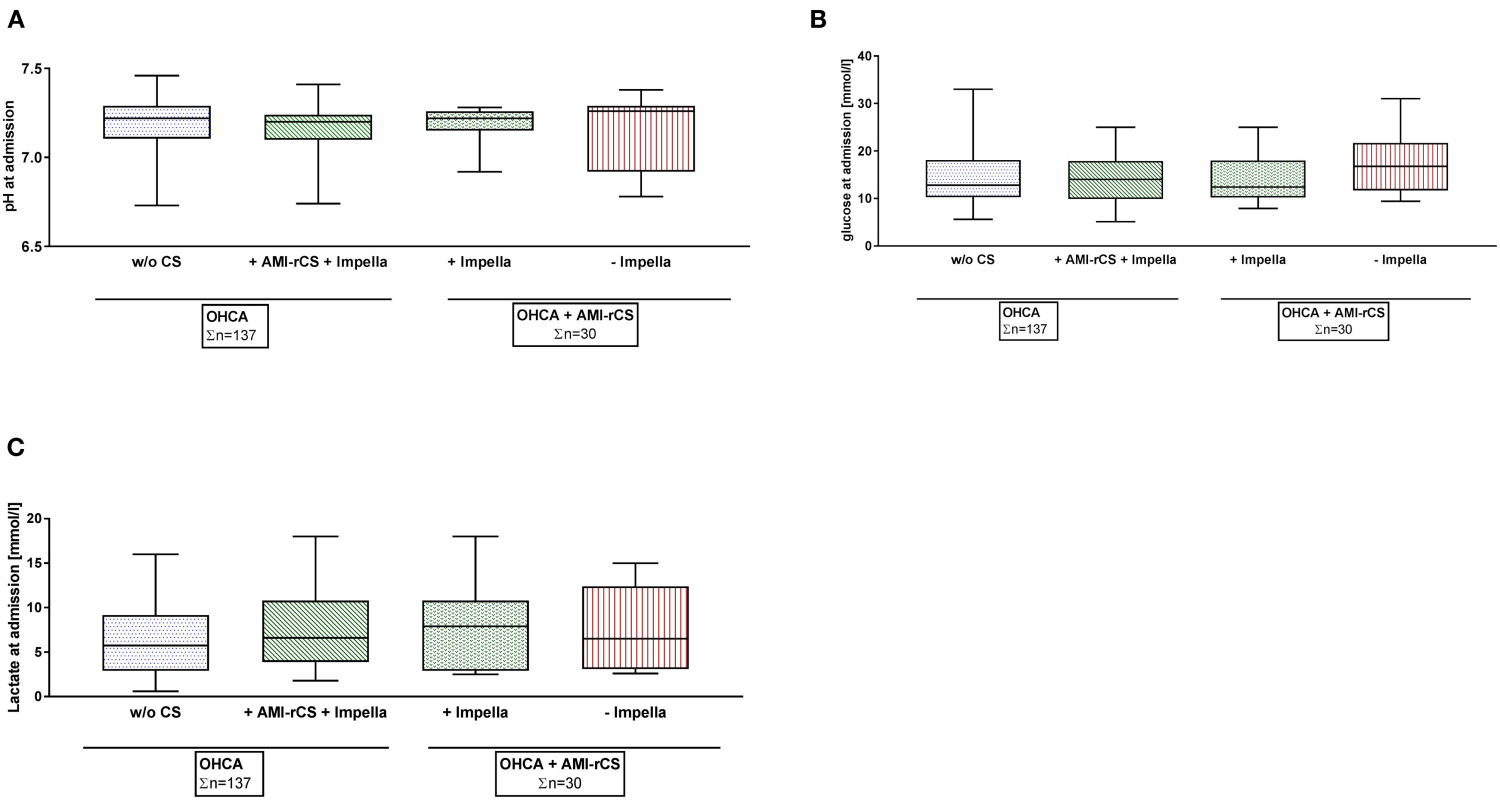
Baseline blood gas analysis of propensity score-matched cohorts. AMI, acute myocardial infarction; CS, cardiogenic shock; OHCA, out-of-hospital cardiac arrest; w/o, without; rCS, refractory cardiogenic shock. **(A)** pH at admission. **(B)** Glucose at admission. **(C)** Lactate at admission.

From both registries, HACURE and HACORE, we identified 47 patients between January 1, 2013 and August 31, 2018 treated with an Impella for AMI–rCS following resuscitation (Figure 1). After 1:1 PS matching, the patients after OHCA with AMI–rCS without Impella (*n* = 15) were included. The patients with AMI without CS complicated by OHCA (*n* = 90) were compared to patients with AMI–rCS and Impella (*n* = 47). The patients with AMI–rCS and active LV unloading with Impella displayed no statistical significance between pH, glucose, and lactate levels at baseline in comparison to patients with AMI–rCS without Impella support (Figure 2). The patients with AMI–rCS after OHCA on Impella support had significantly more vessels treated, longer cumulative stent length, which is explained by standardized complete revascularization in shock at the time of treatment, and higher TIMI risk score. Further patient characteristics are shown in Table 1.

**Table 1:** Baseline characteristics of propensity score-matched cohorts.

	**OHCA**			**OHCA** **+** **AMI–rCS**		
**Variable**	**Without CS,** ***n* = 90**	**+ AMI–rCS** **+ Impella,** *n* **= 47**	***P***	**+ Impella,** *n* **= 15**	**Without Impella,** *n* **= 15**	***P***
Age (years)	67 (57–74)	58 (52–73)	*0.041*	67 (58–78)	66 (55–74)	ns
Length (cm)	176 ± 7	177 ± 7	ns	177 ± 8	177 ± 6	ns
Weight (kg)	82.3 ± 19.3	83.9 ± 13.8	ns	85.1 ± 14.5	67.8 ± 26.9	ns
Gender: male	76 (84%)	38 (81%)	ns	12 (80%)	14 (93.3%%)	ns
Pre-existing disorders						
Smoking	45 (50%)	23 (49%)	ns	5 (33.3%)	7 (46.7%)	ns
Hypertension	58 (64.4%)	28 (59.6%)	ns	13 (86.7%)	7 (46.7%)	ns
Diabetes	23 (25.5%)	8 (17%)	ns	1 (6.7%)	2 (13.3%)	ns
Cardiogenic shock	0	47 (100%)	*<0.001*	15 (100%)	15 (100%)	ns
STEMI	47 (52.2%)	22 (46.8%)	ns	9 (60%)	10 (67%)	ns
NSTEMI	43 (47.8%)	25 (53.2%)		6 (40%)	4 (26.7%)	
Vessels treated (*n*)	2 (1–3)	2 (2–3)	ns	2 (1–2)	1 (1–2)	*0.013*
Cumulative stent length (mm)	27 (18–45)	48 (23–74)	*<0.001*	50 (43–74)	25 (18–50)	*0.024*
Admission lactate (mmol/L)	6.3 ± 3.9	7.3 ± 4.1	ns	7.6 ± 4.8	6.3 ± 3.8	ns
SAPS II score	50 ± 12.4	50.3 ± 9.4	ns	53 ± 14.4	51.8 ± 10.5	ns
CardShock score	4 (3–5)	5 (5–6)	*0.009*	5 (5–6)	5 (5_−_6)	ns
IABP-Shock II score	3 (1–3)	3 (3–3)	*<0.001*	4 (3–5)	4 (3–5)	ns
TIMI risk score	7 (6–9)	8 (7–9)	*0.03*	9 (7–10)	7 (6–9)	*0.047*
In-hospital stay (days)	14.7 ± 7.1	15.6 ± 10.1	ns	17 (4–22)	9 (1–13)	*0.041*

*AMI, acute myocardial infarction; BL, baseline; CS, cardiogenic shock; NSTEMI, non-ST elevation myocardial infarction; rCS, refractory cardiogenic shock; STEMI, ST elevation myocardial infarction*.

### Intensive Care and Safety Outcome

The characteristics of intensive care, MCS, and complications are presented in Table 2. Implementing MCS with Impella in resuscitated, ventilated shock patients in clinical routine practice was associated with <10-min delay of wire crossing over the culprit lesion despite the fact that 68% of cases were performed during on-call time. During ICU stay, all patients were mechanically ventilated. The resuscitation and device characteristics did not significantly differ between groups. The patients after OHCA with AMI–rCS more often required renal replacement therapy compared to patients without rCS. Furthermore, hemolysis was significantly increased in patients after OHCA and AMI–rCS when they were treated with Impella. Bleeding complications occurred significantly more frequently in patients with active left ventricular support with Impella. In the PS-matched cohorts, LV unloading with Impella showed a higher number of patients with a good neurological outcome (CPC ≤2) after 30 days. Vascular ischemic events occurred in both PS-matched cohorts. Due to critical peripheral arterial occlusive disease, vascular intervention was performed in one patient in the OHCA AMI–rCS without Impella group. The other patient received vascular surgery due to critical ischemia after prolonged Impella therapy.

**Table 2:** ICU course and complications of propensity score-matched cohorts.

	**OHCA**			**OHCA** **+** **AMI–rCS**		
**Variable**	**Without CS**,	**+ AMI–rCS**	***P***	**+ Impella**,	**Without Impella**,	***P***
	***n* = 90**	**+ Impella,** ***n* = 47**		***n* = 15**	***n* = 15**	
Bystander CPR performed	68 (75.6%)	37 (78.7%)	ns	11 (73.3%)	10 (66.7%)	ns
Witnessed arrest	79 (87.7%)	39 (83%)	ns	14 (93.3%)	14 (93.3%)	ns
ongoing CPR at admission	5 (5.6%)	6 (65.4%)	ns	0	0	
Out of hospital defibrillation (*n*)	2.9 ± 2.5	3.5 ± 2.9	ns	3.8 ± 3.8	3.7 ± 2.1	ns
Primary rhythm			ns			ns
Asystole	18 (20%)	6 (12.8%)		1 (6.7%)	2 (13.3%)	
Ventricular Fibrillation	72 (80%)	41 (87.2%)		14 (93.3%)	13 (86.7%)	
Time intervals
ROSC (min)	18 (10–30)	23 (10–31)	ns	20 (10–30)	25 (10–35)	ns
Duration puncture to wire crossing (min)	14.3 ± 7.1	24.3 ± 9.9	*<0.001*	21.5 ± 9.9	17.7 ± 5.2	ns
Shock onset to Impella (h)		3 (1.5–4)		3 (2–4)		
Duration of Impella support (h)		89 (46–156)		90 (46–216)		
Impella implantation						
Pre-PCI		28 (59.6%)		8 (53.3%)		
Post-PCI		19 (40.4%)		7 (46.7%)		
LVEDP at the time of PCI	19 ± 6.3 (*n* = 78)	26.7 ± 6.7	*<0.001*	25.5 ± 4.6	25.3 ± 4.5	ns
Bridge to
Deceased during LV support		12 (25.5%)		3 (20%)		
Recovery		34 (72.3%)		12 (80%)		
Durable VAD		1 (2.1%)		0		
RRT during ICU stay	17 (18.9%)	17 (36.2%)	*0.026*	6 (40%)	1 (6.7%)	*0.031*
Hemolysis	0	16 (34%)	*<0.001*	4 (26.7%)	0	*0.032*
Peripheral ischemic complications forcing vascular surgery or intervention	2 (2.2%)	4 (8.5%)	*ns*	1 (6.7%)	1 (6.7%)	ns
Good neurological outcome after 30 days (CPC ≤2)	40 (44%)	24 (51%)	ns	7 (47%)	4 (27%)	ns
GUSTO bleeding			*0.014*			*0.039*
Mild	12 (13.3%)	14 (29.8%)		4 (27%)	2 (13%)	
Moderate	4 (4.4%)	5 (10.6%)		4 (27%)	0	
Severe	0	1 (2.1%)		0	0	

*AMI, acute myocardial infarction; CPC, cerebral performance category; CPR, cardio-pulmonary resuscitation; ICU, intensive care unit; LV, left ventricular; LVEDP, left ventricular end diastolic pressure; PCI, percutaneous coronary intervention; rCS, refractory cardiogenic shock; ROSC, return of spontaneous circulation; RRT, renal replacement therapy; VAD, ventricular assist device*.

### 30-Day Survival in Propensity Score-Matched Groups

**Figure 3:**
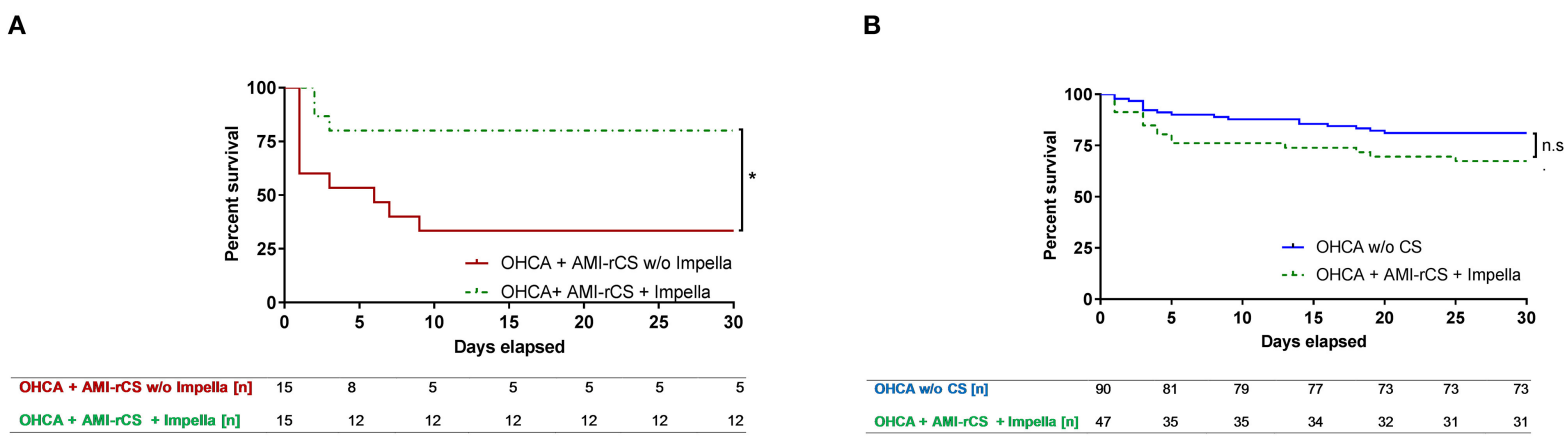
30-day survival after propensity score matching. AMI, acute myocardial infarction; CI, confidence interval; CS, cardiogenic shock; HR, hazard ratio; OHCA, out-of-hospital cardiac arrest; rCS, refractory cardiogenic shock. **(A)** Kaplan–Meier curves of AMI–rCS after OHCA with or without active left ventricular unloading by Impella, *p < 0.05. **(B)** Kaplan–Meier curves of AMI–rCS patients after OHCA without CS or with AMI–rCS supported by Impella.

Compared to resuscitated shock patients without active LV unloading, the patients after OHCA with AMI–rCS on Impella had a significantly higher survival (Figure 3A). During LV support, three patients were deceased due to cardiac deterioration. In the PS-matched groups, patients without LV support had a higher proportion of a cardiac cause of death (*n* = 7 vs. *n* = 3; *p* = 0.024). Furthermore, three additional patients in this group died due to brain damage resulting from extrahospital resuscitation It should be noted that, when the resuscitated patients with AMI–rCS were supported with Impella, they showed no statistical significance for 30-day survival compared to the resuscitated patients without rCS [odds ratio (OR), 0.40; 95% confidence interval (CI), 0.13–1.23; Figure 3B]. In summary, LV unloading with Impella was associated with a markedly lower mortality in AMI–rCS patients after OHCA (OR, 2.67; 95%CI, 1.02–13.66) and HR for 30-day mortality of 0.2 (95%CI, 0.05–0.7).

## Discussion

In our PS-matched analysis comparison to medical treatment only, active LV unloading with an Impella in patients after OHCA with AMI–rCS was associated with a significantly higher survival rate: circulatory support with Impella was a factor for survival until 30 days after hospital admission (OR, 2.67; 95%CI, 1.02–13.66) and HR for 30-day mortality was 0.2 (95%CI, 0.05–0.7). The main conclusion is that our approach of active LV unloading with an Impella micro-axial flow-pump as part of an intra-hospital algorithm (HaCRA) for diagnostic and treatment workflow of patients after OHCA antagonized the severe rCS state, resulting in unexpectedly good 30-day survival rates of around 70%, and the survival rate was comparable to patients after OHCA without rCS.

Cardiac arrest and CS are the main causes of mortality in patients with AMI (1, 16). In previous studies of patients with CS after cardiac arrest, mortality was driven by systolic myocardial dysfunction, hemodynamic instability characterized by reduced cardiac output as well as secondary multiorgan failure and was potentially reversible (17). Despite improved PCI strategies (4, 6) and pre-hospital care (18), the persistently high mortality associated with CS led to the development of several percutaneous MCS devices that are increasingly used in CS. The Impella platform reliably provides hemodynamic stabilization, enhances cardiac output, and reduces end-diastolic wall stress in patients with acute coronary syndrome and STEMI (10, 19).

However, investigations leading to evidence-based assessment of the therapeutic efficacy supporting MCS, especially LV unloading with Impella micro-axial flow pumps, in patients after OHCA complicated by rCS are scarce (20–22). It should be noted that randomized prospective studies using MCS, i.e., Impella or intra-aortic balloon pumps, in patients with rCS, incorporating post-cardiac arrest patients, exhibited a dismal mortality rate of these patients (23, 24). This finding was confirmed by a matched pair analysis applying inclusion criteria IABP-SHOCK II trial (Intra-aortic Balloon Pump in Cardiogenic Shock) (24) in patients with AMI-CS (25).

Besides multiorgan failure and post-cardiac arrest metabolism (3), a contributor to mortality is neurological damage due to anoxic cerebral injury provoked prior to hospital admission (26). Hence, puzzling evidence and ambiguous neurological prognosis of patients after OHCA and rCS at admission result in a reserved approach of MCS implantation.

In our analysis, Impella support was associated in patients after OHCA with AMI–rCS, with a significantly higher survival rate in comparison to conservative treatment. Our approach was associated with comparable mortality rates between patients with OHCA without AMI–rCS and patients with OHCA with additional AMI–rCS supported by Impella. In everyday clinical practice, Impella implantation, as a part of HaCRA, by a multiprofession team was associated with a delay of wire crossing of the culprit lesion below 10 min in comparison to patients without active LV unloading. It should be noted that all groups with applied HaCRA algorithm in this analysis have higher survival rates than previously reported or predicted. In detail, patients with OHCA and AMI–rCS supported with Impella had a better in-hospital survival than predicted by Card Shock score [Card Shock Score: 5 (5, 6), ~70% in-hospital mortality; OHCA AMI–rCS with Impella: 38.5% in-hospital mortality]. In the IMPRESS-in-SEVERE-Shock trial (23), all patients randomized to Impella support had cardiac arrest before implantation (*n* = 24). These patients had a 30-day mortality rate of 46%. In contrast, our analysis of OHCA AMI–rCS patients supported by Impella displayed a 30-day mortality rate of 32%. As opposed to our analysis, in the IMPRESS-in-SEVERE-Shock trial, no standardized algorithm for early diagnosis and treatment of rCS was applied, and Impella implantation was frequently performed after coronary intervention (IMPRESS-in-SEVERE-Shock trial, 80 vs. 39%).

Veno-arterial extracorporeal membrane oxygenation (VA-ECMO) maintains end-organ perfusion and has been conventionally considered after OHCA and rCS. In particular, the use of VA-ECMO during resuscitation as extracorporeal cardio-pulmonary resuscitation (eCPR) recently showed exceptionally good results. In the recently published ARREST trial, early eCPR with VA-ECMO in patients with OHCA and refractory ventricular fibrillation resulted in significant survival to hospital discharge compared with standard therapy (27). Nevertheless, in broader every-day patient cohorts, other groups have reported much lower survival rates sometimes indistinguishable from conventional CPR (28, 29). In rCS without refractory cardiac arrest, however, VA-ECMO increases LV afterload with the consequence of increased filling pressures, pulmonary congestion, and restricted LV recovery (30). Therefore, when treating rCS in stable ROSC after OHCA, we favor the use of the MCS, taking into account its individual characteristics and disadvantages. The DTU-STEMI pilot trial showed that the initiation of active LV unloading by Impella CP in patients with anterior STEMI is feasible and safe (31). Active cardiac support by Impella was associated with a reduced infarct size, increased collateral blood flow to the ischemic myocardium, and reduction of reperfusion injury in a preclinical study (32).

In a recently published analysis of a multicenter registry, 49 patients with acute coronary syndrome-related cardiogenic shock following OHCA were actively supported by Impella (33). The applied treatment protocol, like HaCRA, included an early evaluation of the mechanical circulatory support and prompt coronary angiography. However, the patient characteristics and the post-resuscitation management of the National Cardiogenic Shock Initiative were different to our current analysis. rCS was present in 19 patients (39 vs. 100%), and 19 patients received therapeutic hypothermia after extrahospital resuscitation (39 vs. 100%). The authors displayed a survival rate to hospital discharge of 85.7%.

Further evidence for LV support by Impella in patients with AMI-CS without OHCA will be provided by the ongoing DanGer-SHOCK (Danish–German cardiogenic shock; https://clinicaltrials.gov/ct2/show/NCT01633502?cond=01633502&draw=2&rank=1~NCT01633502) trial (34).

Overall, we strongly believe that HaCRA, as a multidisciplinary early treatment algorithm, supports the early recognition of shock states, initiation of MCS, PCI of the culprit lesion, and mandatory therapeutic hypothermia, resulting in a higher survival rate than that reported and predicted by scores in patients after OHCA complicated by AMI–rCS.

### Limitations

HACURE and HACORE are prospective and observational monocentric registries. Therefore, no randomized control group of the treatment is allocable. HaCRA was performed in a tertiary university hospital setting and was optimized to local conditions. However, applying a standardized protocol, bias cannot be excluded as the decision of indication and the timing of the Impella insertion were done by the physician in charge. This PS analysis included a small series of patients. As a consequence of PS matching with the aim of reducing influencing variables, only a few patients were included in each group. Therefore, the results should be carefully extrapolated owing to potentially unknown covariates and subsequent biases. Furthermore, despite the efforts to form comparable cohorts using a strict post-resuscitation management protocol and PS matching, a possible influence of bias cannot be excluded in this retrospective analysis with a small patient cohort. Overall, the presented results from this non-randomized single-center registry with PS matching have to be considered as hypothesis-generating. However, MCS in rCS and after OHCA is expertise dependent, and patient selection is critical; thus, multi-center studies may be difficult to conduct.

## Conclusion

The results of our analysis suggest that Impella support included in an early intrahospital algorithm (HaCRA) with a multidisciplinary approach and structured diagnostic and therapeutic assessment in patients after OHCA complicated by AMI–rCS and PCI of the culprit lesion is associated with a higher survival rate.

## Data Availability Statement

The raw data supporting the conclusions of this article will be made available by the authors, without undue reservation.

## Ethics Statement

The studies involving human participants were reviewed and approved by ethics committee at Hannover Medical School #3566-2017. The patients/participants provided their written informed consent to participate in this study.

## Author Contributions

J-TS, AS, and JB designed the Hannover Cardiac Unloading Registry. AS, MA, and JB designed the Hannover Cooling Registry. J-TS, MA, J-AB, and UF recruited patients and collected data. J-TS, MA, and J-AB analyzed and interpreted the data. J-TS, MA, JB, and AS wrote the manuscript. JB and AS accurately approved the manuscript. All the authors critically revised and finally approved the manuscript. All the authors agree to be accountable for all aspects of the work and ensure that questions related to the accuracy or integrity of any part of the work will be appropriately investigated and resolved.

## Conflict of Interest

J-TS received travel compensation to congresses from Abiomed. AS and JB received honoraria and research funding from Abiomed. The Department of Cardiology and Angiology of Hannover Medical School is supported by a research grant from Abiomed. The remaining authors declare that the research was conducted in the absence of any commercial or financial relationships that could be construed as a potential conflict of interest.

## Publisher's Note

All claims expressed in this article are solely those of the authors and do not necessarily represent those of their affiliated organizations, or those of the publisher, the editors and the reviewers. Any product that may be evaluated in this article, or claim that may be made by its manufacturer, is not guaranteed or endorsed by the publisher.

## Abbreviations

AMI–rCS: refractory cardiogenic shock owing to myocardial infarction
HaCRA: Hannover Cardiac Resuscitation Algorithm
HACORE: HAnnover COoling Registry
HACURE: HAnnover Cardiac Unloading Registry
LV: left ventricular
MCS: percutaneous mechanical circulatory support
OHCA: out-of-hospital cardiac arrest
ROSC: return of spontaneous circulation.
