# Supplementary material for: Unloading in Refractory Cardiogenic Shock After Out-Of-Hospital Cardiac Arrest Due to Acute Myocardial Infarction—A Propensity Score-Matched Analysis
Source: Front Cardiovasc Med. 2021 Aug 24;8:704312. doi: 10.3389/fcvm.2021.704312 (PMC8421736; doi:10.3389/fcvm.2021.704312)
Supplement: Supplementary file 1 [file Data_Sheet_1.PDF]

Supplemental Material

Methods

Patient treatment

All patients were mechanically ventilated. After PCI, dual anti-platelet therapy with acetylsalicylic acid and prasugrel was administered.(1) Refractory CS was defined as follows: group D and E of the SCAI clinical expert consensus statement, persistence or deterioration of hypotension and/or end organ hypoperfusion (i.e., elevated lactate levels and ≥1 clinical sign of hypoperfusion) despite of catecholamine administration (max. dosing of ≥2 catecholamines) and standard shock therapy.(2, 3) Impella micro-axial flow-pumps were inserted via femoral access under fluoroscopic guidance. Pump placement and anticoagulation were performed as previously described.(4) Weaning of Impella-support was started as soon as possible after hemodynamic stabilization. Before device explantation, the level of LV-unloading was gradually reduced under clinical, echocardiographic, hemodynamic and laboratory evaluation. If LV recovery failed, an institutional heart team evaluated surgical implantation of durable ventricular assist devices (VAD). Hemolysis was defined by typical clinical signs and remarkable hemolytic laboratory parameters (lactate dehydrogenase, cut-off 1000 U/L; free hemoglobin cut-off ≥100 mg/l). Bleeding (mild, moderate, severe) was classified according to the GUSTO-classification. (5) HaCRA in brief, patients after OHCA and/or CS were screened and stabilized in the emergency department by a team of cardiologists and anesthesiologists including: continuation of CPR by an automated compression device in case of ongoing CPR, endotracheal airway management, early determination of cardiac function and valvular disease by transthoracic echocardiography after ROSC, early transfer to the catheterization laboratory or computed tomography if an non-cardiac cause of arrest was assumed, early evaluation of the indication for an active hemodynamic assist device and mandatory therapeutic hypothermia by an intravascular cooling catheter in case of OHCA. Shock severity scores (CardShock score (6), IABP-Shock II risk score (7)) at admission, demographic and laboratory data as well as in-hospital complications are shown in table 1 and 2.

Clinical Follow-up

Patient status was followed-up until 30 days. From the electronic hospital patient data management system data were extracted. In-hospital mortality was recorded online. Consecutive outpatient visits and/or chart review were performed if discharge was performed before 30 days after admission.

Supplemental Table 1: Standardized mean difference

	OHCA +CS	
	+ Impella	w/o Impella
**Variable**	**SMD unmatched (n=70)**	**SMD Matched (n=30)**
age [years]	-0.611	-0.115
bystander CPR performed	0.396	0.143
wittnessed cardiac arrest	0.064	0,001
ROSC [min]	0.067	0,017
primary rhythm	0.131	0.074
myocardial infarction	-0.284	0.113
LVEDP [mmHg] at PCI	0.412	0.141

AMI- Acute myocardial infarction, CPR- Cardio-pulmonary resuscitation, CS- Cardiogenic shock, PCI- Percutaneous intervention, OHCA- Out of hospital cardiac arrest, rCS- Refractory cardiogenic shock, ROSC- Return of spontaneous circulation, SMD- Standardized mean difference, w/o- Without

The standardized mean difference was calculated as previously described by PC Austin. (8)

Supplemental Figure1: 30-day survival before PS-Matching

A

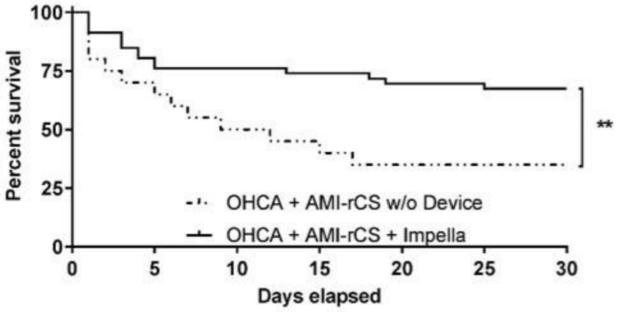

**OHCA + AMI-rCS w/o Device [n]**	23	13	10	8	7	7	7
**OHCA + AMI-rCS + Impella [n]**	47	35	35	34	32	31	31

AMI- Acute myocardial infarction, CS- Cardiogenic shock, OHCA- Out of hospital cardiac arrest, rCS- Refractory cardiogenic shock

A) Kaplan-Meier curves of AMI-rCS after OHCA and with or without active LV unloading by Impella, **$p<0.01$

References

1. Flierl U, Rontgen P, Zauner F, Tongers J, Berliner D, Bauersachs J, et al. Platelet inhibition with prasugrel in patients with acute myocardial infarction undergoing therapeutic hypothermia after cardiopulmonary resuscitation. Thromb Haemost (2016) 115:960-8 doi: 10.1160/TH15-07-0599 [doi].

2. Reyentovich A, Barghash MH, Hochman JS. Management of refractory cardiogenic shock. Nat Rev Cardiol (2016) 13:481-92 doi: 10.1038/nrcardio.2016.96 [doi].

3. Baran DA, Grines CL, Bailey S, Burkhoff D, Hall SA, Henry TD, et al. SCAI clinical expert consensus statement on the classification of cardiogenic shock: This document was endorsed by the American College of Cardiology (ACC), the American Heart Association (AHA), the Society of Critical Care Medicine (SCCM), and the Society of Thoracic Surgeons (STS) in April 2019. Catheter Cardiovasc Interv (2019) 94:29-37 doi: 10.1002/ccd.28329 [doi].

4. Sieweke JT, Berliner D, Tongers J, Napp LC, Flierl U, Zauner F, et al. Mortality in patients with cardiogenic shock treated with the Impella CP microaxial pump for isolated left ventricular failure. Eur Heart J Acute Cardiovasc Care (2018):2048872618757393 doi: 10.1177/2048872618757393 [doi].

5. Mehran R, Rao SV, Bhatt DL, Gibson CM, Caixeta A, Eikelboom J, et al. Standardized bleeding definitions for cardiovascular clinical trials: a consensus report from the Bleeding Academic Research Consortium. Circulation (2011) 123:2736-47 doi: 10.1161/CIRCULATIONAHA.110.009449 [doi].

6. Harjola VP, Lassus J, Sionis A, Kober L, Tarvasmaki T, Spinar J, et al. Clinical picture and risk prediction of short-term mortality in cardiogenic shock. Eur J Heart Fail (2015) 17:501-9 doi: 10.1002/ejhf.260 [doi].

7. Poss J, Koster J, Fuernau G, Eitel I, de Waha S, Ouarrak T, et al. Risk Stratification for Patients in Cardiogenic Shock After Acute Myocardial Infarction. J Am Coll Cardiol (2017) 69:1913-20 doi: S0735-1097(17)30694-0 [pii].

8. Austin PC. An Introduction to Propensity Score Methods for Reducing the Effects of Confounding in Observational Studies. Multivariate Behav Res (2011) 46:399-424 doi: 10.1080/00273171.2011.568786 [doi].
